# Comparison of cumulative viraemia following treatment initiation with different antiretroviral regimens: a real‐life study in Brazil

**DOI:** 10.1002/jia2.25397

**Published:** 2019-11-19

**Authors:** Ana R Pascom, Rosana EGG Pinho, Fernanda Rick, Nazle MC Veras, Filipe de Barros Perini, Mariana V Meireles, Gerson F Pereira, Adele S Benzaken, Vivian I Avelino‐Silva

**Affiliations:** ^1^ Department of Surveillance, Prevention and Control of STIs Ministry of Health of Brazil HIV/AIDS and Viral Hepatitis Brasilia Brazil; ^2^ Tropical Medicine Foundation Heitor Vieira Dourado Manaus Brazil; ^3^ Department of Infectious and Parasitic Diseases Faculdade de Medicina da Universidade de Sao Paulo Sao Paulo Brazil

**Keywords:** HIV, antiretroviral treatment, cumulative viremia, efficacy, antiretroviral regimen, real‐life

## Abstract

**Introduction:**

The relative efficacy of different antiretroviral (ART) regimens has been extensively evaluated in the context of clinical trials, using HIV viral load (VL) measurements at pre‐specified timepoints after ART onset. However, data from real‐life studies using combined longitudinal measurements of cumulative viraemia are scarce. This study aimed to address the independent effect of different ART regimens on HIV cumulative viraemia over the first 12 months after treatment initiation, using programmatic data from the Ministry of Health of Brazil.

**Methods:**

Retrospective cohort study analysing cumulative viraemia under the most frequently used ART regimens in Brazil (tenofovir, lamivudine and dolutegravir (regimen 1); tenofovir, lamivudine and efavirenz (regimen 2); tenofovir, lamivudine and ritonavir‐boosted atazanavir (regimen 3)).

**Results and Discussion:**

We included 112,243 patients >12 years old who received their first ART prescription between January 2014 and August 2017. Univariate analysis indicated that cumulative viraemia was significantly lower in patients receiving regimen 1 as compared with those receiving regimens 2 or 3 (*p*<0.0001 for both pairwise comparisons). In a multivariable analysis adjusted for age, sex, baseline T CD4+ counts and baseline HIV VL, ART regimen persisted with statistically significant effect on 12‐month cumulative viraemia. The model predicted a 45‐unit increase in log_10_ copy‐days/mL cumulative viraemia for regimen 2 as compared with regimen 1, and a 70‐unit increase in log_10_ copy‐days/mL cumulative viraemia for regimen 3 as compared with regimen 1 (95%CI 41 to 49 and 61 to 79 respectively; *p*<0.001 for both comparisons). In models restricted to youths (13 to 24 years old) and female patients, ART regimen had similar effects. ART regimen with dolutegravir in association with a tenofovir‐lamivudine backbone was superior to regimens containing efavirenz or boosted atazanavir in reducing HIV VL, as shown by cumulative viraemia over the first 12 months after treatment initiation. The superiority persisted even after adjusting the analysis for potential confounders.

**Conclusions:**

Our findings could bring direct benefits to patients as suggested by lower viral replication during treatment, lower risk of HIV transmission, and a potential reduction in resistance mutations in the initial 12 months under ART.

## 1 Introduction

The relative efficacy of different antiretroviral treatment (ART) regimens has been extensively evaluated in the context of clinical trials 1, 2, 3. Nevertheless, real‐life studies may still contribute with further knowledge regarding the efficiency and potency of ART regimens in uncontrolled conditions and provide valuable evidence both for individual and programmatic level decisions.

Most studies have used measurements of HIV viral load (VL) at pre‐specified timepoints after ART institution to address antiviral efficacy. However, HIV VL may sometimes fluctuate throughout ART. Factors such as variations in HIV VL assays 4, treatment adherence 5, 6, concurrent infections 7 and immunizations 8 have been implicated in the fluctuations of HIV VL. Even if these problems are absent, the swiftness with which VL decreases can be highly heterogeneous depending on demographic and clinical factors, including ART regimen.

The use of cumulative viraemia measurement as an alternative to single‐timepoint VL has been previously suggested. It has the advantages of incorporating longitudinal measurements of VL, and more broadly depicting the extent of exposure to replicating HIV after the start of ART 9. Cumulative viraemia is defined as the area under the VL curve using individual sequential measurements of VL over a period of time.

Since 2017, dolutegravir has been recommended in association with lamivudine and tenofovir as a preferred regimen for people > 12 years old living with HIV in Brazil. Clinical studies suggest that dolutegravir‐containing regimens are equivalent or superior to alternative ART regimens in both naïve and ART‐experienced patients 10. In this study, we explored the independent effect of the most frequently used ART regimens on HIV VL suppression, using real‐life programmatic data from the Ministry of Health of Brazil. The primary endpoint was HIV cumulative viraemia over the first 12 months after treatment initiation, including subgroup analyses in youths and women.

## 2 Methods

In this retrospective cohort study, we used programmatic data from the Ministry of Health of Brazil, which comprises close to 84% of all people living with HIV referred for ART in the country. We obtained electronic records from two information systems, which gather data on VL and T CD4+ counts performed within the national public health care system, and on every ART prescription.

We identified all patients >12 years old receiving a first ART prescription between January 2014 and August 2017 and persisting with the same ART regimen after ≥12 months. We then selected the patients who had at least two available HIV VL measurements collected at different timepoints. We extracted data on demographics, VL and T CD4+ counts from electronic databases, and included the most recent (baseline) T CD4+ measurement available up to 12 months before treatment initiation.

HIV VL and T CD4+ counts were measured using Abbot Real‐time HIV‐1 with a lower sensitivity of 40 copies/mL and BD MultiTEST CD3/CD8/CD45/CD4 respectively.

The ART regimens that were most frequently employed, and therefore used in our analyses, were: tenofovir, lamivudine and dolutegravir (regimen 1); tenofovir, lamivudine and efavirenz (regimen 2); and tenofovir, lamivudine and ritonavir‐boosted atazanavir (regimen 3).

We obtained VL measurements for individuals in each regimen group at baseline and at every available timepoint up to 12 months after ART introduction. As expected in a real‐life dataset, not all participants had VL measurements consistently performed. Therefore, we used every log_10_‐VL measurement available to determine an individual's cumulative viraemia as previously described 9. In short, cumulative viraemia was estimated as the area under the VL curve, calculated using the trapezoidal rule with all available VL measurements from patients exposed to each ART regimen over the first 12 months of treatment. Higher areas under the VL curve correspond to higher cumulative viraemia, expressed as log_10_ copy‐days/mL.

We explored the effect of each ART regimen on cumulative viraemia in univariate analyses using unpaired T‐tests and in multivariable linear regression models with robust variance estimation adjusted for age, sex, baseline T CD4+ counts and baseline VL. We also included analyses restricted to youths (aged 13 to 24 years old) and women to investigate the effect of the different ART regimens on the cumulative viraemia in these subgroups. Two‐tailed *p*<0.05 were considered statistically significant for all comparisons. All analyses were performed using SPSS (SPSS for Windows 15) and Stata Version 15.1 (StataCorp; StataCorp LP, College Station, TX).

The Scientific Committee approved access to unidentified data from the programmatic register at the Department of Surveillance, Prevention and Control of STIs, HIV/AIDS, and Viral Hepatitis, Ministry of Health of Brazil, with an exemption of informed consent.

## 3 Results and discussion

We included 112,243 patients >12 years old who received their first ART prescription between January 2014 and August 2017 in Brazil. Most were male (72%), of White/Asian (49%) and Black/Mixed (51%) race/ethnicity, and with an overall mean age of 35 years. Before treatment initiation, overall mean T CD4+ count was 457 cells/mm^3^, and mean HIV VL was 4.20 log_10_/mL (Table 1). ART regimens were unevenly distributed in the study population, reflecting national guideline recommendations in the period: 18,830 (17%) patients received regimen 1; 87,896 (78%) received regimen 2; and 5517 (5%) received regimen 3. Age was evenly distributed in the ART regimen groups (Table 1), but the distribution of ART regimens was heterogeneous regarding sex, with a lower percentage of males receiving regimen 3. While 2087 women (7%) received regimen 3, only 3429 (4%) males received the same regimen. We found other differences between males and females in the cohort, particularly in age and race/ethnicity. Mean age at first ART prescription was lower in males (34 vs. 39 years old, *p*<0.0001), and the proportion of black/mixed race was higher in females (55 vs. 50%, *p*<0.001).

**Table 1:** Demographic and clinical characteristics of people living with HIV and receiving a first ART prescription between January 2014 and August 2017, in Brazil, according to regimen group

Characteristics	All patients (N=112,243)	Regimen 1 (N=18,830)	Regimen 2 (N=87,896)	Regimen 3 (N=5517)
Male sex (%)^a^	80,790 (72)	14,129 (75)	63,232 (72)	3429 (62)
Age^b^	35.3 (11.5)	34.7 (11.6)	35.3 (11.5)	37.8 (11.6)
Race/ethnicity^c^				
White/Asian	42,197 (49)	7249 (49)	32,670 (48)	2278 (55)
Black/Mixed	44,563 (51)	7520 (51)	35,152 (52)	1891 (45)
Native Braz.	219 (<1)	44 (<1)	164 (<1)	11 (<1)
Macro‐region^d^				
North	10,977 (10)	1635 (9)	9125 (10)	217 (4)
Northeast	19,995 (18)	3511 (19)	15,676 (18)	808 (15)
Southeast	47,421 (42)	7845 (42)	37,155 (42)	2421 (44)
South	25,717 (23)	4266 (23)	19,756 (23)	1695 (31)
Central West	7798 (7)	1472 (8)	5968 (7)	358 (7)
Baseline T CD4+ counts^e^	457 (368)	456 (317)	457 (380)	461 (327)
Baseline HIV VL (log_10_)^f^	4.20 (1.16)	4.24 (1.16)	4.19 (1.16)	4.16 (1.23)
12‐month cumulative viremia (log_10_ copy‐days/mL)	722.35 (301.54)	689.53 (269.62)	728.03 (306.80)	743.90 (312.25)
ART regimens distribution by age subgroups				
Age				
13 to 17 years‐old (%)	1406	262 (19)	1094 (78)	50 (4)
18 to 24 years‐old (%)	19,865	3770 (19)	15,467 (78)	628 (3)
≥25 years‐old (%)	90,972	14,798 (16)	71,335 (78)	4839 (5)

Regimen 1: tenofovir, lamivudine and dolutegravir; regimen 2: tenofovir, lamivudine and efavirenz; regimen 3: tenofovir, lamivudine and boosted atazanavir. Numerical variables shown as means and standard deviations.

^a^Missing for 21 patients; ^b^missing for 73 patients; ^c^missing for 25,264 patients; ^d^missing for 335 patients; ^e^missing for 12,195 patients; ^f^missing for 4665 patients.

**Figure 1:**
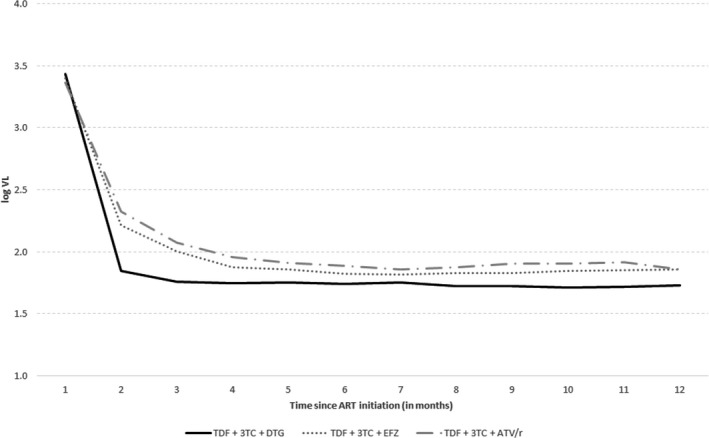
Mean HIV viral load after treatment initiation in people living with HIV and receiving a first ART prescription between January 2014 and August 2017, in Brazil, according to regimen group.

Univariate analysis showed that cumulative viraemia was significantly lower in patients receiving regimen 1 (mean 689.53 log_10_ copy‐days/mL) as compared with those receiving regimens 2 (mean 728.03 log_10_ copy‐days/mL) or 3 (mean 743.90 log_10_ copy‐days/mL; *p*<0.0001 for both pairwise comparisons; Figure 1).

Results from multivariable analysis adjusted for age, sex, baseline T CD4+ counts and baseline HIV VL are presented in Table 2. Older age, female sex and higher baseline HIV VL were associated with higher cumulative viraemia. Higher baseline T CD4+ counts had a borderline association with higher cumulative viraemia. After adjustment, ART regimen persisted with a statistically significant effect on 12‐month cumulative viraemia: the model predicted a 45‐unit increase in log_10_ copy‐days/mL cumulative viraemia for regimen 2 as compared with regimen 1, and a 70‐unit increase in log_10_ copy‐days/mL cumulative viraemia for regimen 3 as compared with regimen 1 (95% CI 41 to 49 and 61 to 79 respectively; *p*<0.001 for both comparisons).

**Table 2:** Effects of ART regimen group on cumulative viremia overall and restricted to youths and to female patients, adjusted for age, sex, baseline VL and baseline T CD4+ counts

	Coefficient	95%CI	*p*‐value
Complete sample			
Regimen group			
Regimen 1 (tenofovir, lamivudine, dolutegravir)	Referent	‐	‐
Regimen 2 (tenofovir, lamivudine, efavirenz)	45.08	40.92 to 49.24	<0.001
Regimen 3 (tenofovir, lamivudine, atazanavir‐r)	70.29	61.44 to 79.14	<0.001
Age (per 5‐year increase)	1.97	0.53 to 3.42	0.007
Female sex	5.10	1.19 to 9.01	0.011
Baseline VL (per log increase)	109.95	102.39 to 117.51	<0.001
Baseline T CD4+ counts (per 100‐cells/mm^3^ increase)	6.77	0.04 to 13.50	0.049
Restricted to young patients 13 to 24 years‐old			
Regimen group			
Regimen 1 (tenofovir, lamivudine, dolutegravir)	Referent	‐	‐
Regimen 2 (tenofovir, lamivudine, efavirenz)	35.37	25.86 to 44.87	<0.001
Regimen 3 (tenofovir, lamivudine, atazanavir‐r)	45.14	21.19 to 69.10	<0.001
Age (per year increase)	1.00	−0.91 to 2.91	0.303
Female sex	−1.21	−12.36 to 9.93	0.830
Baseline VL (per log increase)	118.04	108.23 to 127.84	<0.001
Baseline T CD4+ counts (per 100‐cells/mm^3^ increase)	1.76	−6.97 to 10.49	0.693
Restricted to female patients			
Regimen group			
Regimen 1 (tenofovir, lamivudine, dolutegravir)	Referent	‐	‐
Regimen 2 (tenofovir, lamivudine, efavirenz)	35.37	27.01 to 43.72	<0.001
Regimen 3 (tenofovir, lamivudine, atazanavir‐r)	65.27	49.64 to 80.90	<0.001
Age (per 5‐year increase)	2.85	0.86 to 4.84	0.005
Baseline VL (per log increase)	103.75	86.86 to 120.63	<0.001
Baseline T CD4+ counts (per 100‐cells/mm^3^ increase)	4.13	−9.50 to 17.75	0.553

CI, confidence interval.

In a model restricted to patients aged 13 to 24 years old adjusted for age, sex, baseline T CD4+ counts and baseline HIV VL, regimen 2 was associated with a 35‐unit increase in log_10_ copy‐days/ml cumulative viraemia when compared with regimen 1 (95% CI 26 to 45, *p*<0.001). Regimen 3 was associated with a 45‐unit increase in log_10_ copy‐days/mL cumulative viraemia when compared with regimen 1 (95% CI 21 to 69, *p*<0.001). Interestingly, age, sex and baseline T CD4+ counts were not significantly associated with cumulative viraemia in this subgroup (Table 2).

In a model restricted to female patients adjusted for age, baseline T CD4+ counts and baseline HIV VL, regimen 2 was associated with a 35‐unit increase in log_10_ copy‐days/ml cumulative viremia when compared to regimen 1 (95% CI 27 to 44, *p*<0.001). Regimen 3 was associated with a 65‐unit increase in log_10_ copy‐days/mL cumulative viraemia when compared with regimen 1 (95% CI 50 to 81, *p*<0.001). Similar to our primary analysis, older age was associated with higher cumulative viraemia in this subgroup (Table 2).

Finally, in a model restricted to the ≥24‐year‐old strata, the results were very similar to the model including all participants. We did not find statistically significant interactions between age strata and regimen groups in multiplicative scale (interaction *p*=0.986 and 0.675).

Real‐life studies are essential sources of information for both individual‐based decisions and public healthcare strategies. In this study, we used programmatic data from the Ministry of Health of Brazil to compare the effect of the most frequently used ART regimens on HIV cumulative viraemia, in the first 12 months after treatment initiation. While most existing studies have used HIV VL at pre‐determined timepoints as their primary efficacy endpoints 1, 2, 3, cumulative viraemia combines available VL measurements, capturing the overall trajectory of HIV VL for each patient with greater consistency. Besides, cumulative viraemia potentially translates the effect of lower‐level viraemia under ART on clinical outcomes. For instance, a recent epidemiological study suggested that higher cumulative viraemia is associated with a higher risk of myocardial infarction in people living with HIV 11. Furthermore, cumulative viraemia can be used to estimate the risk of sexual and mother‐to‐child transmission of HIV 12, 13.

We found that the ART regimen containing dolutegravir (+lamivudine‐tenofovir) was associated with significantly lower cumulative viraemia as compared with ART regimens containing efavirenz or boosted atazanavir (+lamivudine‐tenofovir). The favourable effect of the ART regimen containing dolutegravir was observed in both univariate and adjusted analyses, and in analyses restricted to patients aged 13 to 24 years old and to female patients.

Our findings are consistent with previous studies 1, 2, 3, 14, including a previous analysis using real data from the Ministry of Health of Brazil. This study showed a higher percentage of viral suppression at six months after treatment initiation with an ART regimen containing dolutegravir when compared to regimens containing efavirenz, atazanavir or lopinavir 15. The analysis also verified a higher percentage of viral suppression after 12 months of treatment with an ART regimen containing dolutegravir as compared with efavirenz 16. Combined with existing real‐life safety data 17, our study confirms the effectiveness of dolutegravir‐containing regimens to treat HIV infection.

Studies focusing on the efficacy of dolutegravir‐containing regimens to treat teenagers are scarce and generally had small samples 18, 19. In our study, a subgroup multivariable analysis restricted to patients aged 13 to 24 years included 21,271 patients and confirmed the findings shown in the overall sample analysis. This subgroup analysis is reassuring for clinicians who follow youths and provides stronger evidence for the use of dolutegravir as an initial ART, in combination with a double nucleoside/nucleotide backbone. Also, a model restricted to female patients showed that dolutegravir was associated with a lower cumulative viraemia in the first 12 months of treatment when compared to regimens containing efavirenz or boosted atazanavir.

Because our study derives from real‐life programmatic records, we were constrained by what information was available, and had a considerable proportion of missing data. For example, race/ethnicity information was missing for 25,264 (23%) patients; the final multivariable model adjusted for age, sex, baseline VL and baseline T CD4+ counts included 97,386 (87%) of the patients initially included in the study. Even so, the remaining dataset was robust, and we have no evidence that the missing data mechanisms were related to the observed variables. Another potential limitation in our study is the existence of unadjusted confounders. Demographics and clinical characteristics might have influenced the choice of ART regimen and, therefore, have impacted cumulative viraemia – a confounding effect also known as indication bias. We adjusted our multivariable analyses for the main demographic and clinical characteristics that could have influenced the choice of ART regimen. We further opted to exclude regimens using raltegravir, since those would be preferred options for patients with tuberculosis coinfection and pregnant women, conditions possibly associated with higher cumulative viraemia. Additionally, the study period encompasses a recent change in national ART guidelines in Brazil, with the inclusion of dolutegravir as a first‐line option to treat HIV infection in 2017. Hence, it is plausible to infer that the choice of ART regimen reflected current recommendations for most patients and would be less influenced by indication bias.

As opposed to studies in controlled conditions, such as clinical trials and conventional cohort studies, our findings are likely more generalizable, since they included real‐life data, without strict inclusion/exclusion criteria or controlled follow‐up settings.

Our results support recent guideline recommendations by the Ministry of Health of Brazil, which implemented an ART regimen containing dolutegravir as a preferred first‐line ART choice. It also provides encouraging data for the implementation of ART strategies in public health settings in other countries.

## 4 Conclusions

An ART regimen using dolutegravir in association with a tenofovir‐lamivudine backbone was superior to regimens containing efavirenz or boosted atazanavir in reducing HIV VL, as depicted by cumulative viraemia over the first 12 months of treatment, even after adjustments for potential confounders. The results were consistent in youth and female patient subgroups. Our findings have potential implications for clinical practice and could bring direct benefits to patients, as suggested by lower viral replication during treatment, lower risk of HIV transmission, and possible reduction in resistance mutations in the initial 12 months of ART 20.

## Competing interests

All authors declare no competing interests.

## Authors’ contributions

ARPP, REGGP, ASB and VIAS conceptualized study design and data extraction strategy. ARPP, REGGP and VIAS performed data analysis. ARPP, REGGP, FR, NMCV, FBP, MVM, GFP, ASB and VIAS contributed to interpretation of results. ARPP, REGGP, FR, FBP, ASB and VIAS contributed in manuscript writing. All authors revised and approved the final version of the manuscript.
